# Low-Protein Diet Supplemented with Ketoanalogues and Progression of Chronic Kidney Disease in Diabetic Patients: A Multicenter Randomized Controlled Trial

**DOI:** 10.3390/nu18162630

**Published:** 2026-08-12

**Authors:** Ramón Paniagua, Marcela Ávila-Díaz, Julia Nava, Renata Romero-Salas, Juan Carlos H Hernández-Rivera, Oliva Mejía-Rodríguez, Giorgina B Picolli, Mexican Nephrology Collaborative Study Group

**Affiliations:** 1 Unidad de Investigación Médica en Enfermedades Nefrológicas, UMAE Hospital de Especialidades, Centro Médico Nacional Siglo XXI, Instituto Mexicano del Seguro Social, Ciudad de México 06720, Mexico; renataromeros@gmail.com (R.R.-S.); juancarloshhernandezrivera@hotmail.com (J.C.H.H.-R.); 2 Centro de Atención Nutricional Fresenius Kabi, Ciudad de México 11590, Mexico; julnava17@gmail.com; 3 Hospital General de Zona N° 83, Instituto Mexicano del Seguro Social, Morelia 58070, Mexico; 4 Néphrologie, Centre Hospitalier Le Mans, 194 Avenue Rubillard, 72037 Le Mans, France; gpccoli@ch-lemans.fr

**Keywords:** chronic kidney disease, low-protein diets, α-Ketoanalogues, chronic kidney disease progression, type 2 diabetes mellitus

## Abstract

**Background/Objectives**: Low-protein diets (LPD) and very-low-protein diets supplemented with Ketoanalogues (sVLPD) delay initiation of dialysis in non-diabetic patients with chronic kidney disease (CKD). sVLPD is not recommended in patients with type 2 diabetes mellitus (T2DM), and information about LPD supplemented with Ketoanalogues (LPD + KA) is scarce or inconclusive. The objective of this study is to provide further evidence on the benefits and safety of LPD supplemented with KA (LPD + KA) compared with LPD alone in reducing CKD progression in patients with T2DM. **Methods**: T2DM patients in stages 3b-4 of CKD (*n* = 149) were included in a multicenter, randomized, controlled trial and received LPD + KA or LPD, 1 year follow-up. Evaluations were carried out bimonthly. In both groups, protein intake was 0.6 g/kg/day, and the LPD + KA group received KA at the recommended dose. The primary outcome was a decline in eGFR, calculated with Creatinine and with Cystatin C. Secondary outcomes were dialysis requirement, deterioration in nutritional status, hospitalization, morbidity, and mortality. **Results**: During follow-up, protein nitrogen appearance was 0.556 ± 0.119 and 0.576 ± 0.134 g/kg/day in LPD + KA and LPD, respectively (*p* = 0.002). The decrease in eGFR_CysC_ from the baseline was −4.29 ± 0.96 mL/min in LPD + KA and −8.54 ± 2.19 mL/min in LPD (using linear mixed-effects model, *p* = 0.012). eGFR_Cr_ did not show differences. There were no differences in nutrition status, metabolic control, adverse events, hospitalizations, or hospital days. **Conclusions**: Ketoanalogues slow the decline in eGFR_CyC_ but not eGFR_Cr_ in T2DM patients, even without differences in protein intake. LPD + KA may be considered useful and safe; however, due to limitations in sample size and follow-up, as well as observed changes in Cr metabolism, the results warrant confirmation with a more robust design and a longer follow-up period.

## 1. Introduction

The effect of protein intake (PrIn) on the glomerular filtration rate (GFR) and progression of chronic kidney disease (CKD) has been recognized since the early 1980s [1,2]. Results from a significant number of randomized controlled trials (RCTs) support protein restriction as a means of slowing CKD progression and delaying the need for dialysis [3,4]. In the recent KDOQI and KDIGO Clinical Practice Guidelines [5,6], supplemented very low-protein diets (sVLPD), consisting of an intake of 0.3–0.4 g/kg/day and supplemented with a mixture of α-Ketoanalogues (KA) of essential amino acids and some essential amino acids (LPD + KA) (to reach up to 0.6 g/kg/day), have been recommended for non-diabetic patients with CKD. Initiation of these diets is suggested as early as stage 3 CKD [5,6].

Studies comparing low-protein diets (LPD) and sVLPD in diabetic patients have inconclusive results. A meta-analysis found that the original papers have non-comparable designs, and not all have shown that LPD decreases CKD progression [7,8]. The most recent meta-analysis concluded that LPD or VLPD supplemented with KA could safely and effectively prevent kidney function decline and decreased proteinuria. However, it should be noted that the benefit of sVLPD was documented only in patients compliant with the diet (*n* = 11, 0.44 ± 0.11 g proteins/kg/day) compared with non-compliant patients who consumed about 50% more protein (*n* = 9, 0.67 ± 0.21 g proteins/kg/day). Studies on the protective effects of sVLPD on CKD progression in T2DM patients with CKD are limited due to the potential risk of metabolic and nutritional derangement. More extensive RCT studies are warranted to confirm these observations [8]. Observational studies from Italy suggest that the management of T2DM patients should follow similar guidelines to those for non-diabetic patients highlighting the safety of moderate (0.6 g/Kg/day) protein restriction; similar data were reported in a smaller cohort from France [9,10].

Type 2 diabetic patients are probably the most important subset of patients reaching end-stage kidney disease in several settings and are often considered the prototype of fragile, high-comorbidity individuals [11]. The objective of this study is to provide further evidence on the benefits and safety of LPD supplemented with KA (LPD + KA) compared with LPD alone in slowing the progression of kidney damage without compromising the nutritional status of patients with T2DM and CKD.

## 2. Materials and Methods

### 2.1. Study Design

A multicenter, controlled, randomized, open-label clinical trial was conducted in patients with T2DM in CKD stages 3b-4 (eGFR < 44 to >15 mL/min/1.73 m^2^) who were treated in hospitals of the Mexican Social Security Institute and followed up for at least 1 year. Recruitment began in 2019, and follow-up continued until the end of 2020.

### 2.2. Inclusion Criteria

Adult patients with T2DM (>18 years) and stage 3b-4 CKD, who agreed to participate in the study after an invitation from their nephrologist and after being informed by a trained nurse about the RCT, were included in the study. Exclusion criteria: patients unable to express their consent; those with kidney transplantation, cancer, or viral diseases; those with a dialysis-free expectancy of fewer than 3 months; previous treatment with immunosuppressors, hypercalcemia, amino acid metabolism disorders; and intolerance to, or previous treatment with KA. Patients were treated in an outpatient clinic, as recommended by institutional guidelines [12], where nutritional advice allows PrIn to be administered up to 0.8 g/kg/day. However, the use of animal protein sources is not restricted. The prescription of ACEI-ARBs, lipid-lowering drugs, and uric acid control agents is also suggested.

Patients who lost their Social Security number, changed address, or had a different treating physician during follow-up were considered lost to follow-up and excluded from the final analysis. Patients who discontinued the study on their own initiative or due to conditions that impaired regular diet follow-up were considered drop-outs.

### 2.3. Sample Size

The sample size calculation was performed for two independent groups with unequal variances (α = 0.05, β = 0.20), and an assumed difference in eGFR_CyC_ reduction of 4.0 ± 2.0 mL/min/yr between groups at the end of follow-up. The calculation was made using sample size software [13,14,15]. Based on a minimum of 51 patients per arm and the possibility of loss to follow-up, we planned to recruit at least 70 patients per arm.

### 2.4. Intervention

The diet was prescribed by a group of four trained renal dietitians who attended to all patients in a central location without specific assignment (patient–dietitian interaction), considering the patients’ educational level, socioeconomic status, preferences, and accessibility. The attention mechanism includes nutrition information for CKD patients, as well as living workshops with chef direction on food preparation and degustation, held every six months.

### 2.5. Diet Design

Energy according to ideal body weight was 35 kcal/kg in patients with a normal BMI, 30 kcal/kg in overweight patients (BMI > 25 kg/m^2^), and 40 kcal/kg in underweight patients (BMI < 20 kg/m^2^). Lipids accounted for 35% of the total energy intake (2/3 polyunsaturated fatty acids). Carbohydrate intake accounted for 55–60% of total energy intake, with more than 70% of the carbohydrates being complex. The fiber content was 20–25 g per day; the dietary recommendations for sodium chloride were ~5 g/day and potassium 50–60 mEq/day; and phosphate intake was set at 800–1000 mg per day.

Both groups were prescribed a PrIn of 0.6 g/kg/day, which was preferably plant-based. Patients in the LPD + KA group received KA (Ketosteril^®^) (Fresenius Kabi, Deutschland GMBH, BadHomburg, Germany) according to the manufacturer’s recommendations (1 tablet/5 kg of body weight/day, divided into three doses). All other treatments, including insulin and statins, were provided in accordance with institutional guidelines and good clinical practice, and were prescribed by the treating physicians according to their criteria [12].

### 2.6. Randomization

Allocation at each hospital was carried out by the study’s coordinating center using a list of random numbers generated [13] by a collaborator external to the research team, who was not involved in patient recruitment or follow-up. The sequence was blinded for the health team and kept securely. Once it was determined that a patient met the eligibility criteria and agreed to participate, the nephrologist contacted the coordinating center to ascertain the assigned group.

### 2.7. Outcomes

The primary outcome was the rate of decline in kidney function (Δ eGFR/year), calculated using two formulas, one based on Creatinine (eGFR_Cr_) and the other on Cystatin C (eGFR_CyC_). A dual evaluation was necessary because eGFR_Cr_ may overestimate while eGFR_CyC_ may underestimate GFR. Secondary outcomes included the start of dialysis, impairment of nutritional status, adverse events, hospitalizations, and mortality.

### 2.8. Follow-Up

Patients were followed up with scheduled visits every two months for clinical and biochemical evaluation. Blood samples were obtained after an overnight fast, and serum and plasma were separated and kept in frozen aliquots (−70 °C) until assay. The 24 h urine volumes were recorded, and frozen aliquots were stored (−70 °C) until assay. Hemoglobin, hematocrit, and glycated hemoglobin were measured in total blood, and glucose, urea, Creatinine, albumin, total proteins, triglycerides, total cholesterol, HDL cholesterol, calcium, phosphorus, and C-reactive protein were measured in plasma/serum. In 24 h urine: urea and Creatinine were assessed, and serum Cystatin C was measured at baseline 6, 9, and 12 months.

Biochemical parameters were measured on automated equipment using routine techniques. The protein nitrogen appearance normalized by body weight (nPNA) was calculated using the Maroni–Mitch formula [16], and, eGFR was calculated using formulas based on sCr (eGFR_Cr_), according to the CKD-EPI formula, and serum Cystatin C (eGFR_CyC_) [17,18].

### 2.9. Nutritional Evaluations

Nutritional status was monitored using body weight, body mass index [5], subjective global assessment [19], body composition analyzed by electrical bioimpedance [20], and biochemical parameters. As a functional index, hand-grip strength was measured using a dynamometer [21]. Treatment adherence was monitored using the 24 h food intake questionnaire and/or the 3-day food intake diary, as well as nPNA [16]. In the intervention group, adherence to KA use was evaluated by counting tablets and packaging at the end of each month, assuming a consumption of 1 tablet per 5 kg of body weight/day.

### 2.10. Statistical Analysis

Data are shown as means ± standard deviations or standard errors for normally distributed variables, and as medians with interquartile ranges, percentages, or frequencies for non-normally distributed or nominal variables. Per-protocol analysis was conducted. Group differences were assessed using Student’s *t*-test or, when appropriate, nonparametric tests (Squared Chi, Fisher’s, or Moses’s tests). To estimate the decline in GFR over the follow-up period, linear mixed-effects models [22,23] were used, with group and time as fixed effects and age and sex as random effects.

An additional analysis of differences in eGFR decline between groups was also performed, with slopes calculated by least-squares regression for each patient. To analyze the effect of concomitant medication, differences in eGFR regression slopes over time among users and non-users of specific medication were used. Differences in the distribution of adverse events were analyzed using the Squared Chi and the Kolmogorov–Smirnov tests. Metabolic control and nutrition were analyzed for the follow-up period using the linear mixed model. Differences were considered significant when *p* ≤ 0.05 in any statistical analysis. All calculations were performed using SPSS for Windows, version 19.

### 2.11. Ethical Issues

All patients signed informed consent at the time of recruitment. The study was conducted in accordance with the provisions of the Declarations of Helsinki and Tokyo, updated in 2013. The protocol was approved by the National Research and Ethics Committee of the Instituto Mexicano del Seguro Social (registered as code R-2015-785-038 in 18 December 2015, and reapproved using the same code in 13 November 2024), and by the Research Committees of all participating hospitals. The study is registered in National Clinical Trials (NCT03818568, last release 8 June 2021).

## 3. Results

### 3.1. Baseline Data

**Figure 1:**
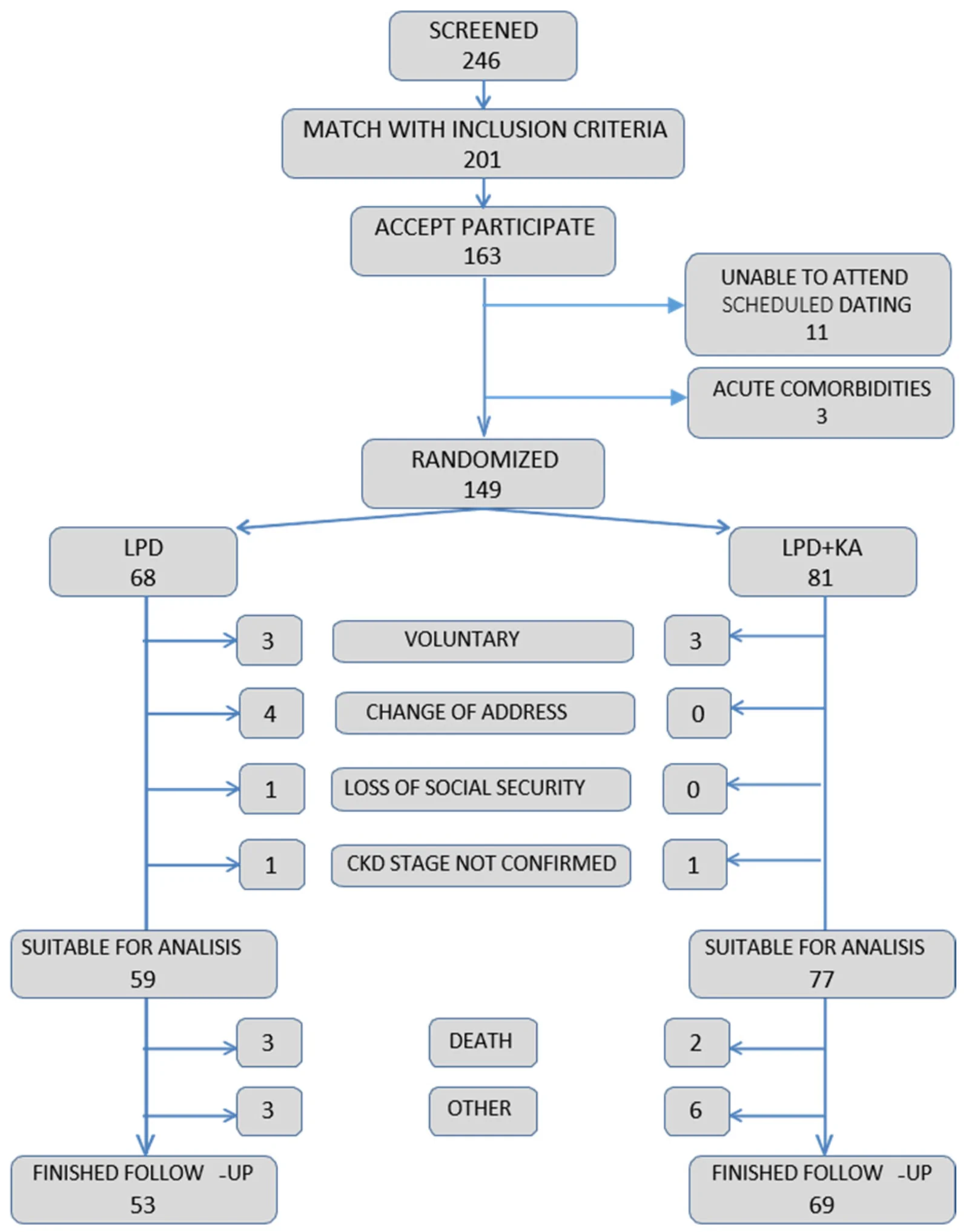
Flow chart of the patients studied from screening to end of follow-up.

The screening, randomization, and follow-up process are illustrated in Figure 1, A total of 122 people completed follow-up: 69 in the LPD + KA group and 53 in the LPD group. This matches the calculated sample size.

Demographic data and baseline values are shown in Tables S1 and S2 in the Supplemental Material. There were no statistically significant differences between groups. At baseline, eGFR_Cr_ did not differ between groups, and eGFR_CyC_ tended to be lower in the LPD + KA group. However, this difference was not significant. eGFR_Cr_ was higher than eGFR_CyC_ in both groups. Patients were already on a PrIn of no more than 0.8 g/kg/day, as recommended by institutional guidelines. Half of the patients were in a suboptimal metabolic control group, with glycated hemoglobin levels above 7.5%.

### 3.2. Outcomes

#### 3.2.1. Compliance

**Figure 2:**
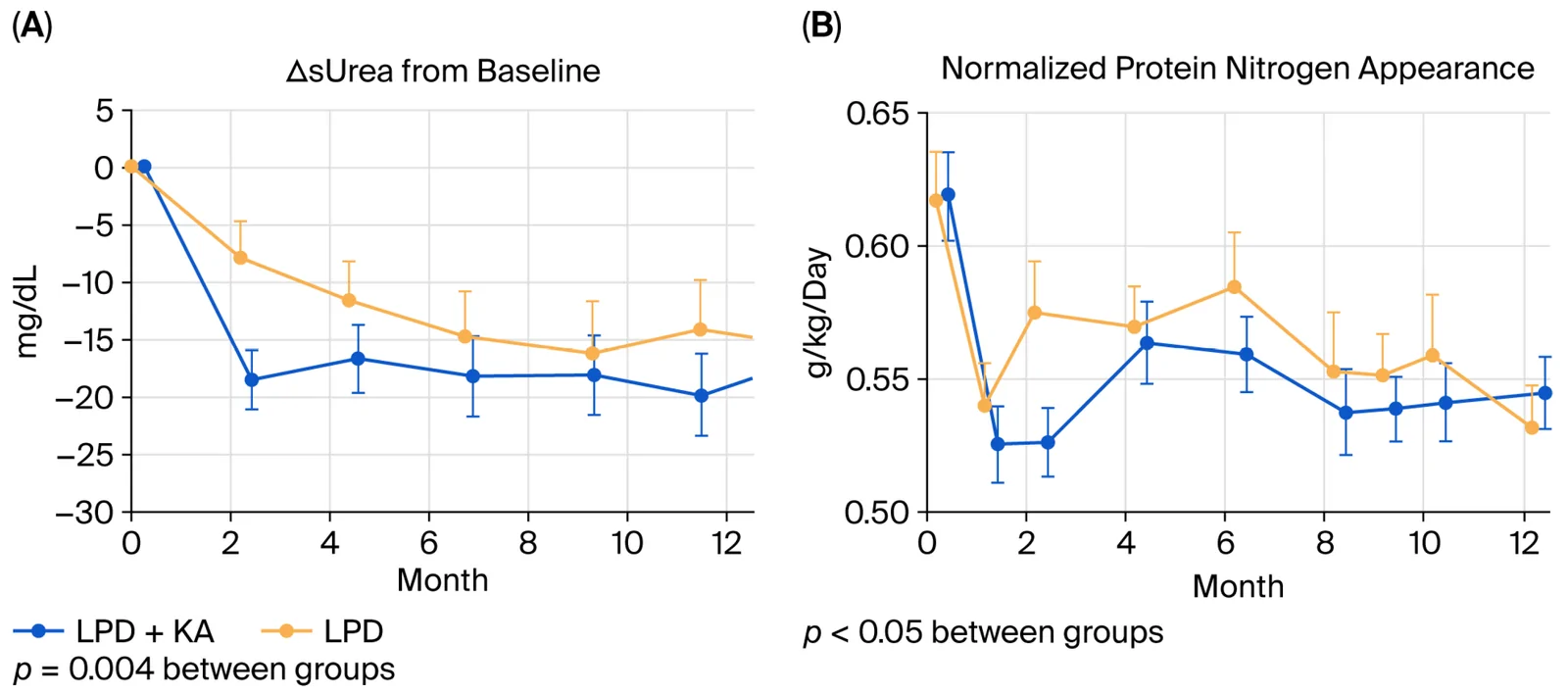
The figure describes adherence to the treatment. (**A**) shows changes in serum urea from baseline values. (**B**) shows nPNA. Variables decreased significantly during follow-up in both groups, with LPD + KA presenting the most notable decline. The effect of Ketoanalogues on the reutilization of protein nitrogen explains differences between groups. Symbols represent means and vertical lines ±1 SEM.

Treatment adherence, as evaluated by nPNA, is shown in Figure 2. In both the LPD and LPD + KA groups, the nPNA was close to the prescribed dose (0.6 g/kg/day) at all time points. Measured nPNA (0.576 ± 0.134 vs. 0.556 ± 0.119, *p* = 0.002) and serum urea concentration were significantly lower in the LPD + KA group throughout the follow-up period (Figure 2). Adherence to the KA prescription, as measured by tablet consumption, was above 95%. No social or demographic variable was associated with adherence to the diet.

#### 3.2.2. Primary Outcomes

**Figure 3:**
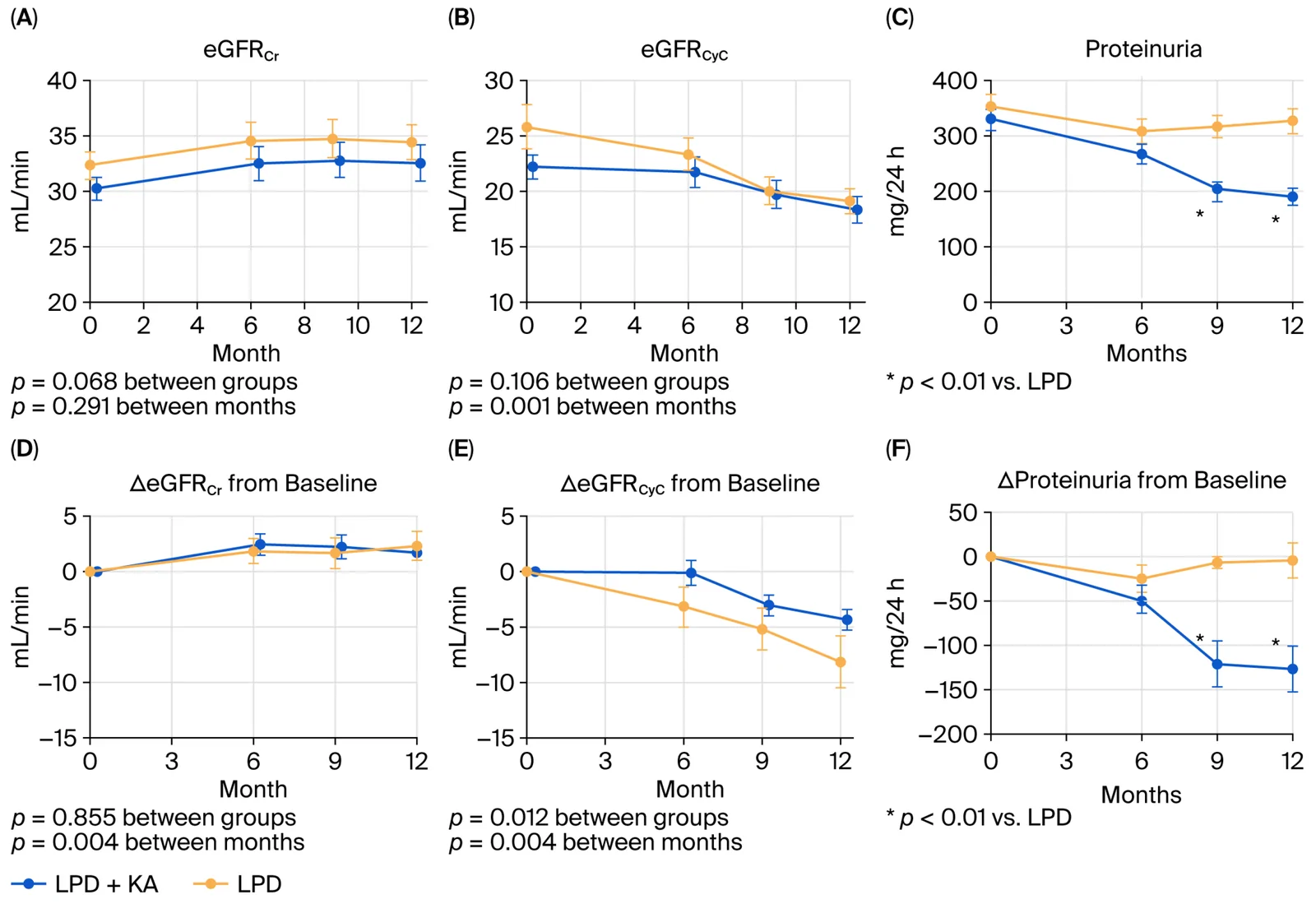
Primary outcomes. (**A**) is the estimated glomerular filtration rate calculated based on serum Creatinine values. (**B**) is the estimated glomerular filtration rate calculated based on serum Cystatin C. (**C**) shows the values for proteinuria. (**D**–**F**) show changes in the same variables relative to baseline values. The decline was more pronounced and significant in ΔeGFRCyC in the LPD group than in the LPD + KA group. Proteinuria and ΔProteinuria decreased more in LPD + KA group. Symbols represent means and vertical lines ±1 SEM—data were analyzed a linear mixed-effects model.

Figure 3A shows the results for eGFR_Cr_; there was a slight increase over time in both groups, but no difference was found between them. Figure 3D shows the results for ΔeGFR_Cr_ from the baseline; no differences were found. eGFR_CyC_ values are shown in Figure 3B of the same figure; in both groups, a decline was observed over time. Even though no significant difference was found, as shown in Table S2, when changes from the baseline were analyzed (using a linear mixed-effects model) (Figure 3E), the decline was significantly lower in the LPD + KA group; values at 12 months were −4.29 ± 0.96 mL/min (mean ± SEM) (mean 95% CI 1.77) for LPD + KA and −8.54 ± 2.19 mL/min (Mean 95% CI 4.27) for LPD. When considering only the final evaluation, a Student’s *t*-test for unequal variances yielded a *p*-value of 0.035.

An additional analysis of differences in eGFR decline between groups was performed, with the slopes were calculated based on least squares for each patient. The results showed marginal but significant differences in eGFR_CyC_ (LPD + KA: −0.34 ± 0.04 mL/min; LPD: −0.72 ± 0.10 mL/min; Student’s *t*-test for unequal variances, *p* = 0.044). Values of proteinuria (Figure 3C) and changes from the baseline (Figure 3F) decreased in the LPD + KA group, but not in the LPD group.

**Figure 4:**
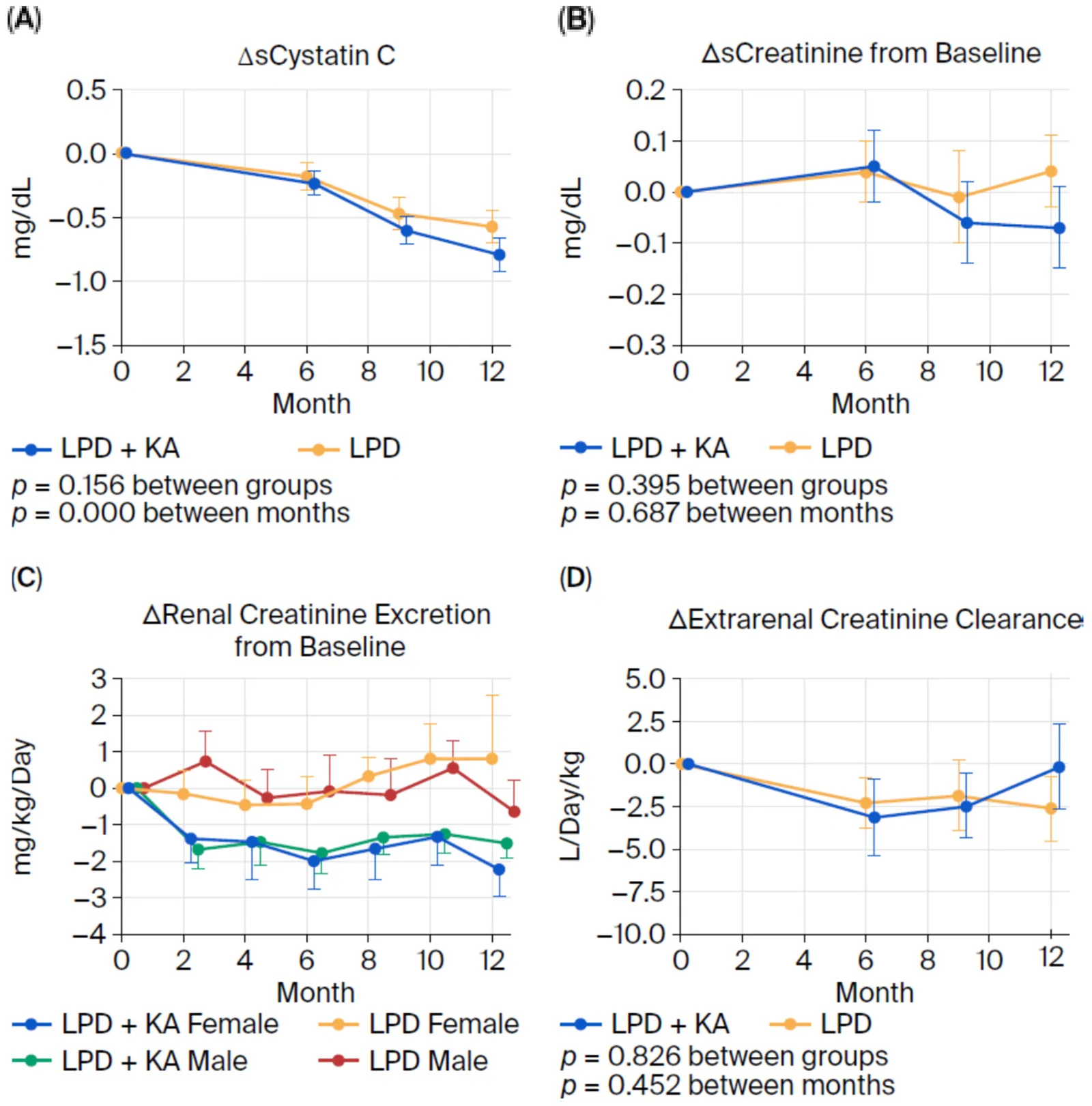
Variables related to Creatinine kinetics were analyzed to explain differences in results when eGFR was estimated using serum Creatinine or serum Cystatin C. (**A**,**B**) show the changes in serum values of Cystatin C and Creatinine, respectively. There was a trend toward reduced levels of the two molecules in both groups, this was more pronounced in Creatinine in the LPD + KA group, but statistical significance was not reached. (**C**) shows significant differences in renal Creatinine excretion, which decreased in both men and women with LPD + KA. (**D**) shows no differences in estimated extrarenal Creatinine clearance or degradation.

The different trajectories between eGFR_Cr_ and eGFR_CyC_ were further analyzed. Figure 4A,B show changes from baseline in serum values of CyC and Cr, respectively. There were no significant differences between groups; however, trends towards decreasing values at 12 months in CyC and Cr were observed in the LPD + KA group. Figure 4C shows changes from the baseline in Cr excretion; a significant decrease was observed in both sexes of the LPD + KA group. Extrarenal Creatinine clearance or degradation remained stable in the two groups. The final eCKD_Cr_ was >9 mL/min in all patients; only one patient in the LPD arm required dialysis due to fluid overload and heart failure.

#### 3.2.3. Secondary Outcomes

##### Metabolic Control

Data on metabolic control and nutrition are shown in Table 1. In both groups, significant improvements were observed in glycated hemoglobin, fasting glucose, total cholesterol, and HDL cholesterol. At baseline, 60.6% of patients in the LPD + KA group and 74.4% in the LPD group used insulin; however, this was reduced in both arms (51.9% and 45.5% in LPD + KA and LPD, respectively; *p* = 0.337). Serum calcium decreased in both groups and was significantly higher in the LPD group (*p* = 0.043, group x month *p* = 0.113). No other differences were seen between groups.

##### Nutritional Status

Neither group showed deterioration in nutritional parameters (Table 1), and both showed a slight improvement in the subjective global assessment score and hemoglobin level. Some parameters did not change during follow-up; these included body weight, body mass index, fat tissue index, serum albumin, lean tissue index (electrical bioimpedance), and hand grip strength. The only significant decline over time was observed in total serum proteins (differences between-months *p* = 0.010, group x month 0.256), but not in serum albumin; the same pattern was observed in both groups.

##### Drug Treatment

All patients received a large number of concomitant medications, which are listed in Table S3 of the Supplemental Material. The table shows the slopes of eGFR_CyC_ decline (mL/min/month) for each medication and compares receivers versus non-receivers. None of these pharmacological treatments influenced the progression of CKD. However, ACE-ARBs, statins, Alopurinol, Bezafibrate, and Thyroid hormones showed an additive effect over LPD + KA.

##### Discontinuation of the Study

Table 2 shows data on dropouts and deaths. There were nine non-fatal dropouts in total, six in the LPD group and three in LPD + KA; five patients died during the period of observation, including three in the LPD group, and two in the LPD + KA group. There were no significant differences in the number or cause of dropouts, in death, or cause of death between the two arms. Table 3 summarizes the rates of the most frequently observed adverse events; their distribution was similar in both arms. The same was observed for hospitalizations.

##### Adverse Events and Hospitalizations

The most frequent adverse events related to the interventions (diet and Ketoanalogues) were hypoglycemia and gastrointestinal disorders, but no difference was seen between groups (Table 3).

There were no significant differences in adverse events; however, events of hypoglycemia events tended to be more frequent in the PLD+KA group, while gastrointestinal disorders were more common in the LPD.

## 4. Discussion

Patients in the LPD + KA group experienced a slower decline in eGFR than those on LPD as assessed by Cystatin C. At the same time, differences were not significant when eGFR was evaluated with serum Creatinine. No differences were found among groups for nutrition variables, adverse events, or hospitalizations.

Adherence is a significant problem in the prescription of LPD or sLPD; it has been found that it improves with better education and younger age [24,25]. Diabetes is also associated with poor adherence, making it challenging to analyze the additional benefit of sVLPD over LPD [26]. In our study, adherence was adequate in both groups. Lower nPNA and urea values in the LPD + KA group were attributed to nitrogen reuse, as observed in balance studies [27]. Although nPNA reflects patients´ adherence to the diet, this technique has a disadvantage: it requires careful 24 h urine collection. Tablet counting also indicates adequate adherence to LPD + KA.

There were differences between eGFR_Cr_ and eGFR_CyC_ in both groups at baseline; discrepancies between techniques have been a cause for concern in previous studies [28]. In some studies, about 30% of patients show differences of up to 15 mL/min/1.73 m^2^ [29]. Our data are within the known limits. The drop in eGFR_CyC_ was faster in the LPD group, consistent with studies in non-diabetic subjects. It is important to note that the difference between groups was evident only in eGFR_CyC_, but not in eGFR_Cr_. The difference lacks a clear explanation, but it is not unexpected. eGFR_Cr_ tends to overestimate GFR, particularly at lower rates, where extrarenal Cr clearance accounts for up to 60% [30]. In the MDRD study, where eGFR was evaluated in conjunction with sCr, no protective effect of sVLPD was observed; instead, an early and steep decline in renal Cr excretion was noted, followed by a gradual decline, which persisted during follow-up and was interpreted as indicative of kidney damage; however, this was not investigated in detail [31]. Similar findings have been reported in at least two other studies; several explanations were identified but not proven [32,33]. In a more recent, well-designed, and well-conducted study, no difference in eGFR_Cr_ was found between LPD and sVLPD, despite the LPD group requiring more dialysis [34], suggesting that eGFR_Cr_ is not very sensitive to detecting differences.

In studies of non-diabetic patients that employed a similar design to this (PrIn 0.6 g/kg/day vs. 0.6 g/kg/day + KA) and measured GFR using methods independent of sCr, significant differences were observed in favor of LPD + KA versus LPD [35,36]. In a recent non-randomized study of diabetic patients treated with PD (0.6 g/kg/day) for 3 months, followed by 9 months of LPD + KA, proteinuria decreased significantly. The decline in eGFR_Cr_ changed from −0.52 mL/min/month to −0.11 mL/min/month over the 9 months of KA intervention. These results align with our study’s data, despite the use of different eGFR assessments [37].

The formulas for calculating eGFR_Cr_ do not account for urinary Cr; however, the lower urinary Cr excretion suggests altered Cr kinetics, rendering eGFR_Cr_ imprecise. Other factors might alter Cr kinetics. A reduction in creatine intake increases creatine synthesis from Creatinine and reduces urinary cCeatinine. Older age and diabetes also contribute to lower excretion; all these factors were present in both groups of our patients [38]. Cr can be transported by organic cation and anion transporters [39,40,41], and polymorphisms in transporter genes influence sCr levels [42]. α-ketoglutarate, other keto acids, and dicarboxylic acids share a transport mechanism with Cr, allowing for competitive inhibition which could explain the observed reduction in Creatinine excretion in the LPD + KA group alone. KA may also compete for organic cation transporter 2, but it has not been sufficiently studied [43]. The reduction in Cr production in the LPD + KA group cannot be explained by loss of muscle mass or extrarenal Creatinine clearance, since lean body mass and hand grip strength did not change during follow-up. Extrarenal Cr clearance also remained stable. Factors influencing CysC synthesis were also considered including, glycemic control, inflammation, and thyroid hormone use [28]. The last two did not differ between groups. Regarding glycemic control, there is a stronger correlation between actual GFR and eGFR_CyC_ than between actual GFR and eGFR_Cr_ in diabetic patients during clamped euglycemia and hyperglycemia [44]. Differences in eGFR_CyC_ between groups did not seem to be influenced by changes in Cystatin C synthesis.

Differences in proteinuria between groups were significant. Groups did not differ in ACEI-ARB use; thus, the nephroprotective effect of LPD + KA could explain this finding. A reduction in proteinuria/albuminuria is a consistent outcome in Ketoanalogues trials. As GFR declines and reductions in proteinuria/albuminuria progress simultaneously, the temporal and causal relationships between these outcomes remain unclear. Our data do not clarify whether a reduction in proteinuria/albuminuria is associated with slower GFR decline, or vice versa.

In previous studies without intervention and RCTs in diabetic patients, sLPD diets were reported to be safe with no deterioration in nutritional status [45,46]. In our study, both diets were safe, and baseline nutritional and metabolic control parameters were similar. Some variables, such as subjective global assessment, hemoglobin, glycated hemoglobin, and serum cholesterol, improved. Variables such as calcium, total proteins, and HDL showed small but significant decreases. We do not have a clear explanation for this data, as diet composition and all other nutritional variables remain unchanged in both groups during follow-up.

Information on the effects of LPD or LPD + KA on mortality is scarce. In a large non-intervention study with a cohort of T2DM-CKD patients, a significant reduction was observed during a 5-year follow-up; however, diet characteristics, precise KA prescription, and biochemical data were not provided [47]. In our study, five patients died of cardiovascular causes without a difference between groups, and in only one case (LPD), it was necessary to start dialysis due to fluid overload and heart failure. There was no difference in the distribution of adverse events per patient or in hospitalizations. Among the adverse events, only hypoglycemic events tended to affect more patients in the LPD + KA group, but no significant complications occurred.

Our study has several limitations. The sample size is relatively small, although randomization worked well. However, despite randomization, more patients were assigned to the LPD + KA group, and an unexpected number left the study for non-medical reasons, with a more pronounced effect in the LPD group. When sample size is small, the risk of non-normal distribution may be a concern. To prevent misinterpretation of the differences in eGFR_CyC_ at the final evaluation, the Moses´ test was performed, with a; *p*-value s 0,019. As an open-label study, patients with LPD may have perceived some disadvantage or disappointment that led them to end the study. To prevent patient–dietitian interaction influencing adherence evaluation, patients were randomly assigned to a dietitian at each visit. Most studies analyze the effects of Ketoanalogues compared to LPD vs. sVLPD. This comparison found a 50% reduction in the hemodynamic effect of protein consumption in addition to Ketoanalogue´s actions. In our study, the intervention may appear minimal because PrIn was the same in both groups, based on plant proteins, and the only difference was the effect of KA. The duration was limited to twelve months from the date of the last patient’s inclusion. Recently, an analysis of appropriate follow-up for studies of GFR decline found that 3 years is the necessary time [48]. Differences in GFR measurement suggest that the gold-standard technique cannot be avoided. This study also has important strengths: It was a multicenter, randomized, controlled trial, and nutritional evaluations were conducted by specifically trained personnel. The study included only patients with T2DM, without selection based on safety or therapeutic compliance. It is important to note that the SGLT2 inhibitors currently used and recommended, such as GPL-1, and nsMRAs were used only in selected patients at our institution (IMSS) during the time period for which the study was designed and approved. Studies combining these new treatments with sLPD are not available.

## 5. Conclusions

The use of Ketoanalogues slows the decline in eGFR in T2DM patients when eGFR is based on Cystatin C and not Creatinine. The effect was significant even with similar protein intake and limitations in sample size and follow-up. The results also suggest that changes in Creatinine metabolism are secondary to low GFR and, probably, to KA use. The diet was well tolerated, and adherence was high. LPD + KA may be considered useful and safe, without deterioration in nutrition, hypercalcemia, or derangement of metabolic control. The results warrant confirmation with a more robust design and a longer follow-up period, as well as a deeper analysis of Creatinine metabolism.

## Figures and Tables

**Table 1:** Values of nutrition parameters, mineral metabolism, glucose metabolism, lipid metabolism, and inflammation during follow-up. LPD + KA and LPD had reductions in the percentages of score 2 and increments in score 1 of Subjective global assessment. Both groups showed significant improvements in glycated hemoglobin, fasting glucose, cholesterol, HDL cholesterol, and insulin requirements with no differences between them. A reduction in serum calcium concentration was observed in both groups, but remained higher in LPD than in LPD + KA (*p* = 0.041). Total proteins values decreased from baseline in both groups with no differences between them. Differences between groups and months were analyzed by a linear mixed model.

	Month								
	Group	0	2	4	6	8	10	12	*p*
*n*	LPD + KA/LPD	77/59	76/59	74/56	73/55	73/54	72/53	69/53	
Body weight (kg)	LPD + KA	71.95 ± 12.01	69.69 ± 11.96	69.18 ± 11.55	70.07 ± 12.35	70.21 ± 12.09	70.12 ± 12.35	69.85 ± 11.78	0.998
	LPD	72.69 ± 14.20	71.29 ± 13.87	71.68 ± 13.84	71.21 ± 12.40	71.58 ± 12.53	71.56 ± 11.97	72.39 ± 12.46	
Body mass index (kg/m^2^)	LPD + KA	28.82 ± 4.09	27.97 ± 4.16	27.96 ± 4.19	28.24 ± 4.53	28.20 ± 4.47	28.15 ± 4.14	28.40 ± 4.20	0.994
	LPD	28.82 ± 4.62	28.43 ± 4.42	28.61 ± 4.44	28.52 ± 3.80	28.29 ± 3.86	28.56 ± 3.95	28.96 ± 4.11	
Subjective global assessment (score) 1	LPD + KA	77.92	87.01	89.19	88.41	90.48	88.06	93.44	0.045
1	LPD	78.33	89.47	92.98	83.64	93.62	94.00	95.65	
2	LPD + KA	22.08	12.99	10.81	11.59	9.52	11.94	6.56	
2	LPD	21.67	10.53	7.02	16.36	6.38	6.00	4.35	
Serum albumin (G/dL)	LPD + KA	4.01 ± 0.49	4.02 ± 0.38	4.08 ± 0.31	4.00 ± 0.36	4.01 ± 0.35	3.98 ± 0.36	3.94 ± 0.37	0.179
	LPD	4.13 ± 0.42	4.10 ± 0.38	4.05 ± 0.34	4.02 ± 0.35	4.04 ± 0.34	4.01 ± 0.32	3.96 ± 0.42	
sTotal protein (g/dL)	LPD + KA	7.07 ± 0.74	6.99 ± 0.59	7.01 ± 0.62	6.88 ± 0.52	6.72 ± 0.56	6.67 ± 0.58	6.62 ± 0.61	0.000
	LPD	7.15 ± 0.60	7.06 ± 0.64	6.96 ± 0.62	6.91 ± 0.62	6.77 ± 0.59	6.73 ± 0.49	6.67 ± 0.71	
Hemoglobin (g/dL)	LPD + KA	12.02 ± 2.03	11.40 ± 2.17	11.90 ± 1.78	12.15 ± 2.46	12.64 ± 1.81	12.14 ± 1.94	12.14 ± 2.30	0.000
	LPD	12.71 ± 2.59	11.98 ± 2.34	12.01 ± 2.86	12.60 ± 2.72	12.97 ± 3.06	13.07 ± 3.18	13.74 ± 2.66	
Lean tissue index (kg/m^2^)	LPD + KA	13.42 ± 2.81	12.88 ± 2.73	12.97 ± 2.70	13.16 ± 2.89	13.18 ± 2.87	13.04 ± 2.92	13.29 ± 3.14	0.991
	LPD	13.23 ± 2.66	13.34 ± 2.83	13.37 ± 2.63	13.82 ± 2.68	13.36 ± 2.67	13.55 ± 3.16	13.86 ± 2.79	
Fat tissue index (kg/m^2^)	LPD + KA	14.46 ± 5.00	14.13 ± 5.28	13.97 ± 5.35	14.11 ± 5.43	14.00 ± 5.31	14.15 ± 5.32	14.08 ± 5.30	0.999
	LPD	14.88 ± 5.64	14.35 ± 5.55	14.43 ± 5.49	13.90 ± 4.71	14.14 ± 4.93	14.21 ± 5.20	14.27 ± 4.86	
Hand grip strength (kg)	LPD + KA	20.77 ± 6.44	21.44 ± 6.55	21.21 ± 6.57	21.40 ± 6.10	21.26 ± 5.74	20.77 ± 6.56	20.75 ± 6.70	1.000
	LPD	22.39 ± 7.94	22.71 ± 7.71	22.22 ± 7.55	22.59 ± 7.82	22.58 ± 7.67	22.47 ± 7.96	23.38 ± 8.24	
Serum calcium (mg/dL)	LPD + KA	9.11 ± 0.66	8.98 ± 0.46	9.02 ± 0.79	8.94 ± 0.54	8.91 ± 0.89	9.29 ± 0.59	8.85 ± 0.58	0.000
	LPD	9.36 ± 0.95	9.15 ± 0.64	9.05 ± 0.52	9.04 ± 0.54	9.28 ± 0.57	9.36 ± 0.75	8.91 ± 0.57	
Serum phosphorus (mg/dL)	LPD + KA	4.28 ± 0.70	4.10 ± 0.64	4.02 ± 0.76	4.00 ± 0.64	3.85 ± 0.71	4.18 ± 0.60	3.89 ± 0.65	0.174
	LPD	4.19 ± 0.98	3.94 ± 0.61	4.01 ± 0.60	3.95 ± 0.62	4.05 ± 0.73	4.00 ± 0.56	3.91 ± 0.61	
Glycated hemoglobin (%)	LPD + KA	7.44 ± 1.75	7.29 ± 1.37	7.15 ± 1.32	7.10 ± 1.43	7.01 ± 1.38	6.69 ± 1.08	6.57 ± 1.15	0.000
	LPD	7.72 ± 1.72	7.43 ± 1.49	7.06 ± 1.75	6.93 ± 1.19	6.84 ± 1.34	6.65 ± 0.88	6.22 ± 0.97	
sGlucose (mg/dL)	LPD + KA	122.49 ± 51.57	114.36 ± 46.72	117.94 ± 43.15	115.86 ± 46.49	112.99 ± 36.28	115.10 ± 48.85	117.43 ± 57.07	0.185
	LPD	123.55 ± 46.31	112.29 ± 47.58	109.49 ± 43.13	107.11 ± 36.04	114.35 ± 42.44	104.89 ± 49.86	108.94 ± 45.28	
sCholesterol (mg/dL)	LPD + KA	192.92 ± 66.62	175.55 ± 46.15	173.61 ± 38.54	173.88 ± 46.60	171.44 ± 41.83	162.26 ± 38.28	158.64 ± 38.15	0.000
	LPD	187.70 ± 51.34	175.44 ± 39.77	170.41 ± 36.74	167.78 ± 35.00	162.23 ± 33.12	158.00 ± 28.21	154.45 ± 29.32	
sHDL cholesterol (mg/dL)	LPD + KA	38.38 ± 13.38	35.96 ± 10.56	35.93 ± 8.67	34.63 ± 8.82	34.83 ± 8.36	33.79 ± 8.82	32.55 ± 10.32	0.039
	LPD	38.24 ± 11.77	37.80 ± 11.54	36.63 ± 9.63	36.85 ± 11.13	35.79 ± 10.65	34.48 ± 9.40	35.81 ± 10.82	
Triglycerides (mg/dL)	LPD + KA	190.79 ± 130.52	200.90 ± 144.01	179.35 ± 64.59	181.64 ± 102.69	176.08 ± 72.21	181.00 ± 116.77	174.86 ± 86.23	0.420
	LPD	187.15 ± 87.09	169.27 ± 72.83	161.15 ± 75.54	165.66 ± 93.89	156.13 ± 73.79	156.60 ± 73.47	152.43 ± 65.40	
C-Reactive protein (mg/dL)	LPD + KA	5.46 ± 6.85	6.87 ± 4.36	6.23 ± 1.76	5.67 ± 6.71	6.18 ± 1.91	7.76 ± 6.65	7.50 ± 11.69	0.848
	LPD	5.77 ± 5.17	6.48 ± 2.24	7.10 ± 2.59	6.42 ± 10.12	8.67 ± 5.17	8.07 ± 7.28	5.73 ± 4.56	

**Table 2:** Dropouts and deaths were similar in both groups. The *p*-values refer to the distribution of events across groups.

Dropouts					
		LPD	LPD + KA	Total	*p*
Total Dropouts		9	5	14	0.731
Non-fatal dropouts		6	3	9	0.580
Cause					
	Hip fracture	2	1	3	
	Trauma accident	0	1	1	
	Neuropathy	1	0	1	
	Cholecystectomy	1	0	1	
	Obstructive uropathy	1	0	1	
	Coronary revascularization	1	0	1	
	Peritoneal dialysis (heart failure and, fluid overload)	0	1	1	
Deaths		3	2	5	0.652
Cause					
	Acute myocardial infarct	2	2	4	
	Sudden death	1	0	1	

**Table 3:** The *p*-values refer to the distribution of events across groups.

Adverse Events							
	LPD + KA			LPD			
Adverse Event	Patients	Events	Event/pt · yr	Patients	Events	Event/pt · yr	*p*
Hypoglycemia	22	30	0.28	7	10	0.12	0.095
Urinary tract infection	9	14	0.13	9	11	0.13	0.287
Upper respiratory tract infection	28	38	0.35	22	35	0.33	0.692
Eye cataract, vitrectomy, conjunctival bleeding	6	8	0.06	1	1	0.01	0.248
Hypertension	7	11	0.10	2	5	0.06	0.187
Cardiovascular	7	9	0.08	3	3	0.04	0.548
Trauma, accident	13	15	0.14	5	7	0.08	0.211
Gastrointestinal disorders	25	37	0.35	29	41	0.45	0.441
Dermatitis	7	7	0.07	3	3	0.03	0.292
Abdominal surgery	4	4	0.04	4	4	0.05	0.488
Other	24	30	0.27	14	17	0.20	0.727
Total events	58	177	1.63	47	130	1.50	0.457
Hospitalizations	21	30	0.28	7	9	0.11	0.161

## Data Availability

The data presented in this study are available on request from the corresponding author due to privacy reasons since the data underlying this article are sensitive health data and cannot be shared publicly.

## Supplementary Materials

The following supporting information can be downloaded at: https://www.mdpi.com/article/10.3390/nu18162630/s1.

## Abbreviations

The following abbreviations are used in this manuscript:

PrIn	Protein Intake
LPD	Low-Protein Diet
KA	Ketoanalogues
LPD + KA	Low-Protein Diet Plus Ketoanalogues
sVLPD	Very-Low-Protein Diet Supplemented with Ketoanalogues
CKD	Chronic Kidney Disease
RCT	Randomized Controlled Trial
T2DM	Type 2 Diabetes Mellitus
eGFR	Estimated Glomerular Filtration Rate
eGFR_Cr_	Estimated Glomerular Filtration Rate Based on Serum Creatinine
eGFR_CyC_	Estimated Glomerular Filtration Rate Based on Serum Cystatin C
nPNA	Protein Nitrogen Appearance Normalized by Body Weight
