# Supplementary material for: Low-Protein Diet Supplemented with Ketoanalogues and Progression of Chronic Kidney Disease in Diabetic Patients: A Multicenter Randomized Controlled Trial
Source: Nutrients. 2026 Aug 12;18(16):2630. doi: 10.3390/nu18162630 (PMC13516023; doi:10.3390/nu18162630)
Supplement: Supplementary file 1 [file nutrients-18-02630-s001.zip › nutrients-4401079-supplementary.pdf]

Supplemental Material

Mexican Nephrology Collaborative Study Group*

Collaborators:

Renal nutrition team: Abril Gutiérrez-Espinosa, Araceli Aguilar-Gamiño, and Natalí Sánchez-Godoy;

Monitoring team: Florencia Castro, Patricia Hernández, Diana Morán, and Miguel-Ángel Trejo;

Biochemical analysis: Santa-Adelina Prado-Espinosa and Miriam Cruz-Gómez;

Dabase management: Alejandro Hinojosa-Rojas.

Nephrologists in IMSS Mexico City Centers: Eunice Rivera-Luna, H.G.Z. 24; Emilia Cantoral-Farfán, H.G.Z. 32; Bárbara Ulibarri, HGR 1. Carlos MacGregor; Adrián Ramírez-Cárdenas, H.G.Z. 2A, Francisco del Paso y Troncoso; María-Antonia Camarillo-Castillo, H.G.Z. 8; Daniel Román-Márquez, H.G.Z. 27; Begoña Ilabaca-Avendaño, H.G.Z. 1A, Venados. Nephrologists in other IMSS places: Efraín Arredondo-Santoyo, Nancy-Argelia Valdez-Carreón, H.G.Z. 83, Morelia, Mich; María-Azucena Soto, H.G.Z. 194, Naucalpan, Edo. México.

Table S1. Social and demography characteristics

		LPD+KA	LPD	All	p
n		77	59	136	
Gender (Male, %)		59.00%	61.00%	60.00%	0.520
Marital status					0.944
	Unmarried	3.80%	1.70%	2.90%	
	Married	70.50%	70.00%	70.30%	
	Widow	17.90%	20.00%	18.80%	
	Divorced	3.80%	5.00%	4.30%	
	Free Union	3.80%	3.30%	3.60%	
Education					0.403
	Less than elementary	3.80%	3.30%	3.60%	
	Elementary	43.60%	50.00%	46.40%	
	Junior High School	17.90%	13.30%	15.90%	
	High School	15.40%	8.30%	12.30%	
	Non-university technical career	9.00%	5.00%	7.20%	
	University	10.30%	20.00%	14.50%	
Economical income[1]					0.424
	<200	2.60%	3.30%	2.90%	
	200-300	66.70%	53.30%	60.90%	
	300-500	24.40%	31.70%	27.50%	
	>500	6.40%	11.70%	8.70%	

[1]Income is expressed in USD by month. p values are the significance of distribution between groups analyzed by Squared Chi test.

Table S2. Clinical and biochemical variables

		LPD+KA			LPD			Total			
		Mean		ED	Mean		ED	Mean		ED	p
	n	77			59			136			
Non-Biochemical	Age (Year)	67.15	±	8.05	68.05	±	6.84	67.54	±	7.53	0.490
	Systolic blood pressure (mmHg)	146.26	±	25.16	143..85	±	20.92	145.21	±	23.36	0.551
	Diastolic blood pressure (mmHg)	78.60	±	12.05	78.93	±	11.63	78.75	±	11.83	0.871
	Weight (kg.)	71.96	±	12.01	72.69	±	14.20	72.28	±	12.95	0.742
	Height (cm)	157.86	±	9.48	158.37	±	9.46	158.08	±	9.44	0.755
	Body Mass Index (kg/m2)	28.85		4.14	28.89		4.43	28.87		4.26	0.959
Biochemical	Hemoglobin (g/dL)	12.02	±	2.03	12.71	±	2.59	12.31	±	2.30	0.177
	Glycated Hemoglobin (%)	7.44	±	1.75	7.72	±	1.72	7.55	±	1.73	0.357
	Serum Glucose (mg/dL)	122.50	±	51.57	123.55	±	46.31	122.95	±	49.18	0.901
	Serum Creatinine (mg/dL)	2.33	±	0.73	2.19	±	0.71	2.27	±	0.72	0.266
	BUN (mg/dL)	41.28	±	11.98	38.84	±	22.34	40.22	±	17.24	0.411
	Serum Total protein (g/dL)	7.07	±	0.74	7.15	±	0.60	7.11	±	0.68	0.527
	Serum Albumin (g/dL)	4.01	±	0.48	4.13	±	0.42	4.07	±	0.46	0.132
	Serum Cystatin C (mg/dL)	2.59	±	0.68	2.52	±	0.76	2.61	±	0.72	0.180
	C-Reactive protein (mg/dL)[4]	4.10	±	3.48	4.22	±	3.40	4.16	±	3.43	0.283[5]
	Serum Triglycerides (mg/dL)	190.79	±	130.52	187.15	±	87.09	188.18	±	113.06	0.853
	Serum Total Cholesterol (mg/dL)	192.92	±	66.62	187.70	±	51.34	190.62	±	60.21	0.617
	Serum HDL Cholesterol (mg/dL)	38.38	±	13.38	38.24	±	11.77	38.31	±	12.65	0.948
	Serum Total Calcium (mg/dL)	9.11	±	0.66	9.36	±	0.95	9.21	±	0.81	0.072
	Serum Phosphorus (mg/dL)	4.28	±	0.70	4.19	±	0.98	4.24	±	0.83	0.530
	eGFRCKDEPI (mL/min)	31.70	±	12.03	33.60	±	12.07	32.53	±	12.04	0.359
	eGFRCysC (mL/min)	22.26	±	9.44	26.29	±	15.50	24.01	±	12.55	0.061
	nPNA (g/kg/day)	0.62	±	0.15	0.62	±	0.14	0.62	±	0.14	0.948

No differences between groups were seen. [1]$eGFR_{CKDEPI}$= estimated Glomerular Filtration Rate by serum creatinine level. [2]eGFRCysC= Estimated Glomerular Filtration Rate by serum Cystatin C level. [3]nPNA= Normalized Protein Nitrogen Appearance. For CRP: [4]=Median ± Interquartile range. [5]= Mann-Withney "U" test.

Table S3. Effect of specific medication on the slop eGFR decline (evaluated by Cystatin C)

		Total				LPD+KA				LPD				User vs non-user	LPD+KA vs LPD
Medication		%	Mean		SD	%	Mean		SD	%	Mean		SD	p	p
ACEs or ARBs	No	16.67	-0.249	±	0.782	17.81	-0.068	±	0.547	14.29	-0.543	±	1.036	0.441	0.033
	Yes	83.33	-0.422	±	0.686	82.19	-0.309	±	0.675	85.71	-0.563	±	0.680		
Insulin	No	19.38	-0.434	±	0.923	24.66	-0.358	±	1.028	12.50	-0.630	±	0.594	0.550	0.085
	Yes	80.62	-0.384	±	0.643	75.34	-0.235	±	0.489	87.50	-0.550	±	0.657		
Statins	No	48.84	-0.344	±	0.607	46.58	-0.312	±	0.573	51.79	-0.381	±	0.769	0.243	0.015
	Yes	51.16	-0.441	±	0.784	53.42	-0.225	±	0.728	48.21	-0.753	±	0.116		
Alopurinol	No	52.71	-0.423	±	0.768	53.42	-0.231	±	0.627	51.79	-0.682	±	0.871	0.471	0.021
	Yes	47.29	-0.361	±	0.625	46.58	-0.305	±	0.698	48.21	0.430	±	0.524		
Bezafibrate	No	56.59	-0.412	±	0.712	76.71	-0.294	±	0.676	71.43	-0.578	±	0.737	0.519	0.025
	Yes	43.41	-0.340	±	0.680	23.29	-0.174	±	0.603	28.57	-0.516	±	0.730		
Pentoxyfilline	No	75.97	-0.364	±	0.722	75.34	-0.211	±	0.704	76.79	-0.560	±	0.840	0.435	0.100
	Yes	24.03	-0.489	±	0.640	24.66	-0.434	±	0.467	23.21	-0.562	±	0.729		
Thyroid hormones	No	89.15	-0.433	±	0.695	86.30	-0.324	±	0.629	92.86	-0.565	±	0.752	0.255	0.049
	Yes	10.85	-0.072	±	0.703	13.70	0.101	±	0.747	7.14	-0.504	±	0.347		

Slopes of $eCFR_{Cyc}$ decline over the follow-up period were obtained for each patient. Differences in slopes between users and non-users of each specific medication were calculated using Student's t-test. [1]Angiotensin-converting Enzyme Inhibitors. [2]Angiotensin Receptor Blocker
